# Cytokine Therapy in Bladder Cancer: Mechanisms, Efficacy, and Future Prospects

**DOI:** 10.3390/cimb47040278

**Published:** 2025-04-15

**Authors:** Hayden J. Oyler, Layne G. Bruton, Austin J. Maher, Darien A. Yu, Nicholas W. Shely, Mark R. Wakefield, Yujiang Fang

**Affiliations:** 1 Department of Microbiology, Immunology & Pathology, Des Moines University, West Des Moines, IA 50266, USA; hayden.oyler@dmu.edu (H.J.O.); layne.g.bruton@dmu.edu (L.G.B.); nicholas.w.shely@dmu.edu (N.W.S.); 2 Department of Surgery, School of Medicine, University of Missouri, Columbia, MO 65211, USA; ajmnkm@missouri.edu (A.J.M.); dayqdd@missouri.edu (D.A.Y.); wakefieldmr@health.missouri.edu (M.R.W.); 3 Ellis Fischel Cancer Center, School of Medicine, University of Missouri, Columbia, MO 65211, USA

**Keywords:** bladder cancer, cytokine therapy, immunotherapy, pro-inflammatory cytokines, anti-inflammatory cytokines, engineered cytokines, fusion proteins

## Abstract

Cytokine therapy is a rapidly evolving field in bladder cancer research, with treatments designed to enhance immune responses, improve targeting, and promote tumor cell recognition and elimination. This review explores pro-inflammatory cytokines, anti-inflammatory cytokines, engineered cytokines and fusion proteins, and combination therapies. Challenges include risks of toxicity, immune suppression, and the potential for promoting metastasis. Despite these obstacles, the potential successes of cytokine therapies highlight the importance of continued investigation into their use for developing safe, effective, and minimally invasive treatments for bladder cancer.

## 1. Introduction

Bladder cancer is the sixth most common cancer in the United States, with 83,190 new cases and 16,840 deaths estimated for 2024 [1,2]. Worldwide, it is the ninth most frequently diagnosed cancer, with 524,305 new cases estimated in 2019. While the absolute number of cases has increased, the age-standardized incidence rate has remained relatively stable [3]. Bladder cancer is classified into two groups based on the extent of invasion: non-muscle-invasive bladder cancer (NMIBC) and muscle-invasive bladder cancer (MIBC). NMIBC is confined to the bladder mucosa and submucosa, while MIBC extends into the underlying muscularis propria and beyond [4]. While treatment approaches differ, cystoscopy and urine cytology remain the gold standard for diagnosing both types of bladder cancer [5]. First-line treatment for NMIBC is Bacillus Calmette–Guérin (BCG) intravesical therapy or radical cystectomy in patients who are unresponsive to BCG therapy. MIBC requires radical cystectomy with lymph node dissection as the primary treatment, followed by additional systemic treatments and testing due to its higher risk of metastasis [4]. Immunotherapy represents a newer and rapidly developing approach to treating bladder cancer. While it has been used for autoimmune disorders, its most significant impact has been in oncology, where it has revolutionized cancer treatment [6]. As a result, much of the current research is dedicated to exploring its potential in cancer therapy [7].

**Figure 1:**
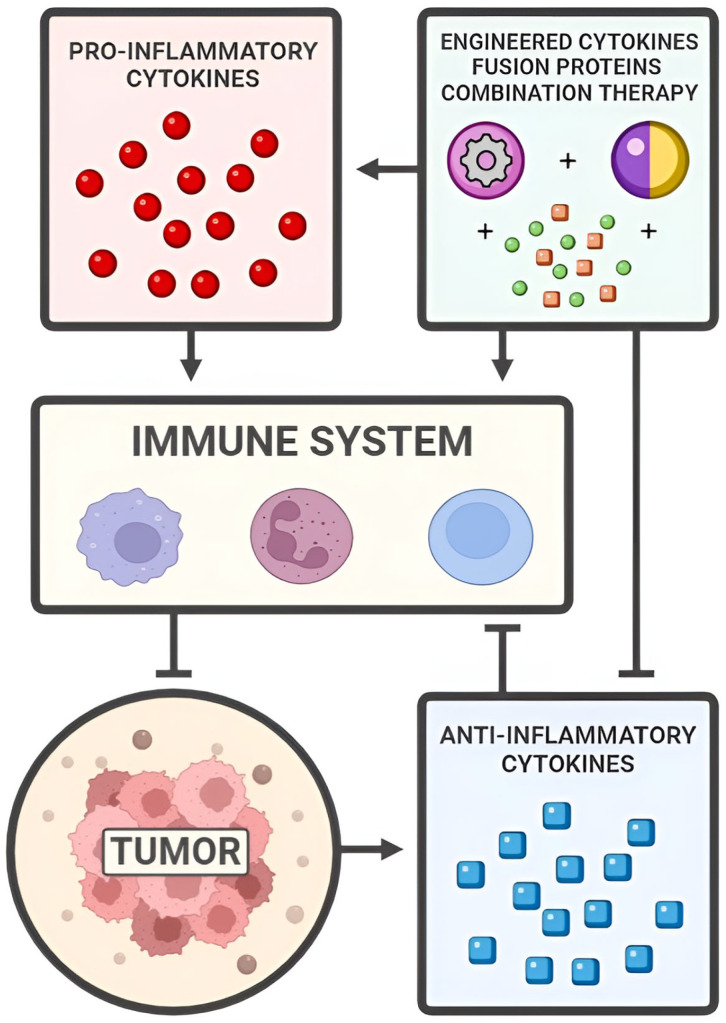
Summary of the mechanisms employed by pro-inflammatory cytokines, anti-inflammatory cytokines, engineered cytokines, fusion proteins, and combination therapies.

Immunotherapy aims to harness and enhance the body’s immune system to combat various diseases by targeting specific cells and modulating the immune response [8]. It can be classified as either passive or active: passive immunotherapy delivers immediate immunity via pre-formed components like monoclonal antibodies and CAR T cells (chimeric antigen receptor T cells), while active immunotherapy stimulates the immune system to generate a response through vaccines and immune checkpoint inhibitors [9,10]. Cytokine therapy is a branch of immunotherapy involving the administration of one or more cytokines to enhance immune activity. Cytokines are signaling proteins that regulate complex pathways involved in immune and inflammatory responses. Examples include the stimulation of B cell antibody isotype switching, differentiation of T helper cells (Th cells) into Th-1 and Th-2 subtypes, and the activation of phagocytic macrophages [11]. Many cytokines are pleiotropic, inducing distinct differentiation effects based on the target cell receptor. IL-2 (interleukin-2), for example, activates both cytotoxic and regulatory T cells (CTL and Tregs), which have opposing functions [12]. Major cytokine families include interleukins (ILs), interferons (IFNs), tumor necrosis factors (TNFs), granulocyte–macrophage colony-stimulating factors (GM-CSFs), and chemokines [13]. This review examines the current state of cytokine therapy in bladder cancer, focusing on its molecular mechanisms and outcomes, as summarized and illustrated in Figure 1. Given their pleiotropic nature, cytokines have many overlapping functions, but for this paper, we primarily categorize them as being either pro-inflammatory or anti-inflammatory.

## 2. Materials and Methods

**Figure 2:**
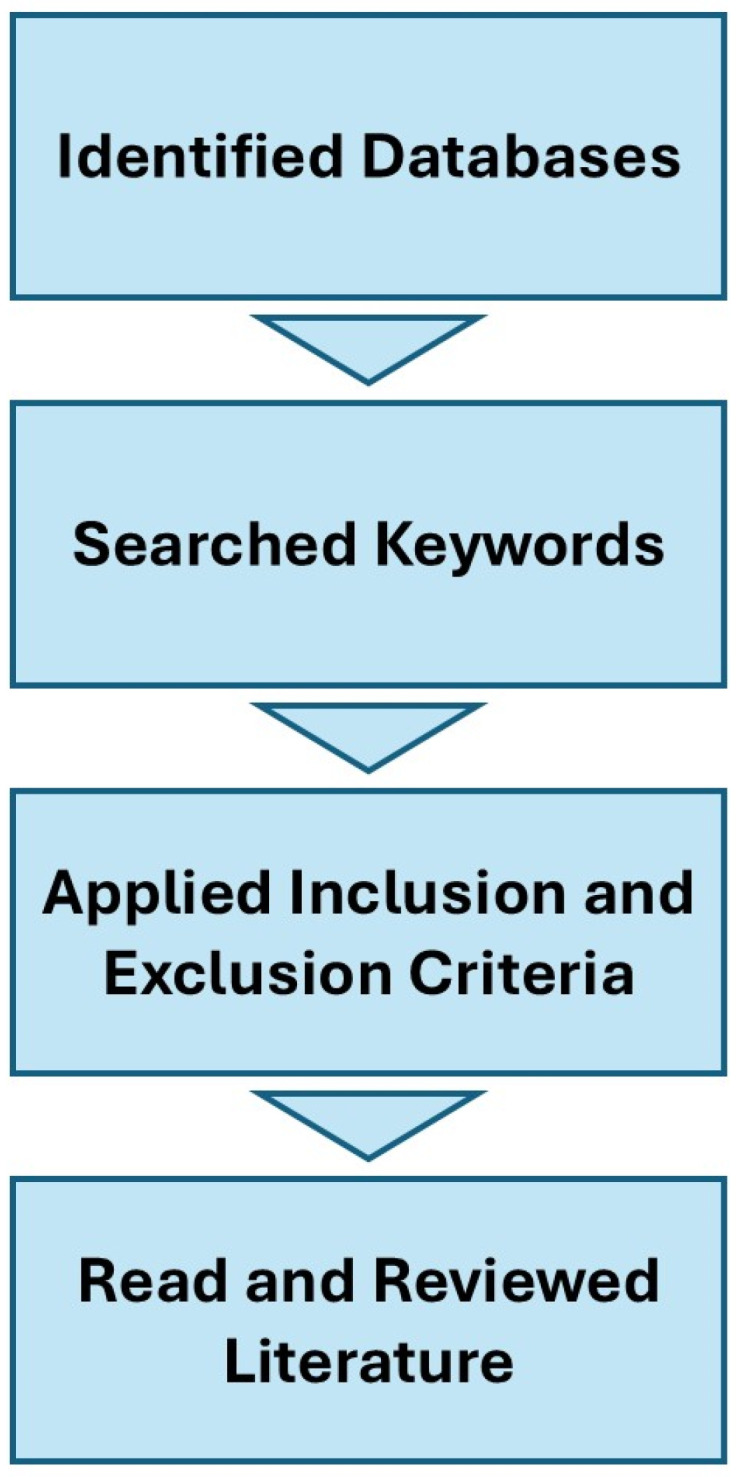
Flowchart outlining the literature review process for writing this manuscript.

For this review paper, a literature search was conducted in PubMed, Google Scholar, Scopus, EBSCO, NCBI, Cochrane Library, Web of Science, and National Library of Medicine from 28 January 2025 to 28 February 2025. Keywords used in this search were cytokines, inflammatory cytokines, anti-inflammatory cytokines, cytokine antagonists, fusion proteins, engineered cytokines, combination therapy, cytokine therapy, immunotherapy, mechanism, biochemistry, molecular, bladder cancer, tumor proliferation, treatment, cytokine therapy, inflammation, immune response, IL-2, IL-8, IL-12, TGF-β (transforming growth factor-β) IL-10, IL-4, EGF (epidermal growth factor), CD40 (cluster of differentiation 40), IL-15, and PD-1 (programmed death receptor-1). Inclusion criteria consisted of English-language publications, clinical and preclinical studies, systematic reviews, and meta-analyses, all from peer-reviewed journals. Exclusion criteria included non-peer-reviewed articles, irrelevant topics, non-English studies, and older publications (>25 years), unless they provided foundational knowledge. References from selected studies were manually screened to identify additional relevant articles. Data were analyzed to assess the efficacy, safety, and potential of cytokine immunotherapies in treating bladder cancer, and studies were screened independently by two reviewers. The study selection process is outlined in Figure 2.

## 3. Pro-Inflammatory Cytokines

Pro-inflammatory cytokines such as IL, IFN, and TNF are produced by various immune cells to trigger inflammation and regulate immune responses [14]. They play an active role in directing immune cells to sites of infection and injury for inflammation and tissue repair, as well as the tumor microenvironment (TME) to promote the elimination of tumor cells [15]. IL-12, for example, promotes the differentiation of naïve CD4+ cells into type 1 T helper cells (Th1) and enhances CD8+ cytotoxic T cells (CTLs) and natural killer (NK) cells in their anti-tumor responses [16]. Given their role in immune activation, pro-inflammatory cytokines are promising candidates for stimulation in treating cancer [17]. Cytokines like IL-2, IL-8, and IL-12 have shown potential in bladder cancer therapy and remain important areas of research.

### 3.1. IL-2

IL-2 is primarily produced by CD4+ T cells following antigen presentation by antigen-presenting cells (APCs) [18]. Under normal physiological conditions, IL-2 binds to the high-affinity IL-2 receptor on T cells to promote their proliferation and differentiation, particularly into CD8+ cytotoxic T cells and regulatory T cells, which help carry out the immune response and target cancer cells [19]. Originally developed as a tuberculosis vaccine, BCG enhances IL-2 expression following intravesical administration, where it attaches to and is internalized by cancer cells. This process allows BCG to act as a pathogen-associated molecular pattern (PAMP), triggering APC activation, IL-2 production, and an immune response against tumor cells [20,21].

BCG therapy has shown greater success in immune activation when combined with IL-2 as opposed to BCG therapy alone [22]. Additionally, IL-2 complexes (IL-2cs) were found to reduce tumor size in mouse orthotopic urothelial carcinoma (UC) cell line MB49 and mouse bladder transitional cell carcinoma cell line MBT-2. In the MB49 cell line, IL-2c acted by decreasing the expression of Tregs, which promote immunosuppression [23]. Dosing presents a complication in IL-2 treatment in bladder cancer and contributes to its limited therapeutic index. High doses can be toxic, while low doses can promote immunosuppression via Treg activation, both of which must be considered while developing treatments involving IL-2 [24].

Recent research into the IL-2 prodrug bempegaldesleukin (BEMPEG) addresses the cytotoxicity concerns associated with traditional IL-2 therapies by increasing CD8+ T cells while leveraging IFN-γ and TNF-α to reduce Tregs in tumors [25]. When administered intravenously in combination with Nivolumab, BEMPEG has demonstrated effectiveness in reducing the tumor size, achieving a 35% objective response rate (ORR), with minimal side effects in patients with advanced or metastatic urothelial carcinoma [26]. This combination therapy appears to be the most effective approach, as intravesical IL-2 instillations alone did not enhance therapeutic outcomes in patients with marker lesions, which were expected to boost IL-2 efficacy [27].

### 3.2. IL-8

IL-8 is a chemokine involved in inflammation through the activation and recruitment of neutrophils to the site of inflammation. It binds to its receptors, CXCR1 and CXCR2, on local cells to initiate neutrophil chemotaxis, degranulation, and respiratory burst [28]. IL-8 is significantly overexpressed in invasive and high-grade urothelial bladder cancer tumors as it can promote tumor progression through several mechanisms [29]. The CXCR 2 receptor of IL-8 promotes tumor angiogenesis and allows for leukocytes to enter the tumor microenvironment [30]. Neutrophil recruitment is particularly problematic, as tumor-associated neutrophils drive the epithelial–mesenchymal transition (EMT) in cancer cells, leading to metastasis [31]. Additionally, IL-8 induces the transcription of cell proliferation genes and the downregulation of tumor suppressor genes via cascade signaling of the MAPK (mitogen-activated protein kinase) pathway and the JAK2 (janus kinase 2) and FAK (focal adhesion kinase) pathways, respectively [32,33].

Bladder cancer tumors secrete IL-8 to recruit neutrophils, which, rather than aiding the immune system, target CD8+ T cells meant for tumor destruction. This neutrophil manipulation highlights the need to understand tumor–neutrophil crosstalk to develop more effective bladder cancer treatments [34]. Given its pro-tumor activity, IL-8 is an ideal target for inhibition in cancer therapy. A recent study found that arsenic exposure can lead to IL-8 overexpression via the phosphorylation of human epidermal growth factor receptor 2 (HER2), promoting migration and EMT while activating ERK (extracellular signal-regulated kinase), AKT, and STAT3 (signal transducer and activator of transcription 3) signaling pathways to increase cancer stem cell markers such as CD44 in bladder epithelial cells. The same study found that genistein effectively inhibited migration and EMT in the human uroepithelial cell line SV-HUC-1 by preventing HER2 phosphorylation, blocking downstream signaling pathways, and suppressing IL-8 expression [35].

Since IL-8 expression is a known predictor of bladder cancer prognosis, elevated IL-8 levels in urine have been strongly suggested as a valuable biomarker for urothelial carcinoma [36]. Beyond this association, however, research is limited on IL-8 inhibition, presenting an opportunity for further exploration of this cytokine in cancer treatment.

### 3.3. IL-12

IL-12 is produced by APCs and plays a key role in inflammation. It binds to the IL-12Rβ2 receptor, activating the JAK-STAT4 pathway, which promotes IFN-γ production by T and NK cells. This activation increases the cytotoxicity and proliferation of NK cells, promotes the differentiation of Th1 cells, and stimulates a pro-inflammatory response [37]. Because of its physiological role in promoting the immune response, treatment options aim to enhance IL-12 activity or restore it from a suppressed state.

IL-12 alone has shown limited efficacy, leading to the development of CS/IL-12, an intravesical immunotherapy combining chitosan and IL-12 for bladder cancer treatment [38]. Chitosan, a mucoadhesive polymer derived from chitin, helps IL-12 penetrate the urothelium by binding to the negatively charged bladder mucosa and increasing epithelial permeability [38,39,40,41]. In an orthotopic bladder tumor mouse model, CS/IL-12 treatment resulted in 88% long-term survival, with all mice treated in three rounds becoming tumor-free [39]. Despite this success, further research is limited, partly due to IL-12’s dose-dependent toxicity [42].

To overcome this issue, another study explored using red blood cell-derived extracellular vesicles (RBCEVs) to deliver IL-12 minicircle plasmids intratumorally. This approach reduced the tumor growth in MB49 mouse bladder cancer cells while enhancing the pro-inflammatory response with enriched CD4+ and CD8+ T cells. Notably, RBCEV delivery showed no signs of kidney or liver toxicity [43]. Another recent study in human trials investigated a combination therapy using M9241 (NHS-IL12) with avelumab. The participants received subcutaneous M9241 every four weeks and intravenous avelumab every two weeks. Although the IL-12 levels increased along with CD8+ T cell and NK cell proliferation, the treatment demonstrated limited efficacy. Despite the lack of significant clinical benefit in patients with advanced urothelial carcinoma, the therapy was well-tolerated, underscoring the need for further research and development to improve IL-12-based treatments [44].

## 4. Anti-Inflammatory Cytokines

There are many cytokines secreted by the human body that can have either positive or negative effects on the body, depending on their concentration. Anti-inflammatory cytokines are produced by cells to reduce inflammation, promote lymphocytic tumor infiltration, and expand the production and reactivation of T cells and NK cells that attack cancer cells [15,45]. In cancer, tumor cells release chemokines that attract tumor-associated macrophages (TAMs) to the tumor site. TAMs then proliferate and secrete anti-inflammatory cytokines to fight malignant cells [46]. Cancer cells eventually develop resistance, leading to excessive anti-inflammatory cytokine production, which suppresses inflammation and enhances immune evasion, allowing tumors to grow and metastasize [47,48,49]. To counter this, therapies use cytokine antagonists to block cytokine–receptor interactions by binding to the cytokine or its receptor, reducing its effects. This approach has successfully mitigated the impact of excessive anti-inflammatory cytokines [49,50].

### 4.1. TGF-β

TGF-β is an important anti-inflammatory cytokine in regulating cell proliferation. In early tumor development, TGF-β suppresses tumor progression by activating kinase inhibitors that induce cell cycle arrest and apoptosis. During the later stages of tumorigenesis, however, cancer cells often acquire mutations that enable them to evade these inhibitory effects. In such cases, TGF-β remains present but becomes ineffective at halting tumor growth and may even promote it by reducing inflammation [51,52]. TGF-β also plays a significant role in immune evasion by inhibiting NK cells. It downregulates CD16+, a receptor essential for NK cell cytotoxicity, and can drive NK cells toward a pro-angiogenic CD16- phenotype, further supporting tumor progression [45,53]. Additionally, TGF-β suppresses T cells and macrophages, promotes angiogenesis, and induces enzymes that degrade the extracellular matrix, facilitating metastasis [54]. Excessive TGF-β secretion has been strongly linked to tumor growth, invasion, and poor prognosis in bladder cancer, making it a compelling target for immunotherapy [55].

One cytokine antagonist that has been popular for treating TGF-β is Galunisertib, a small molecule kinase inhibitor designed to bind to TGF type 1 receptors and block the signaling pathway of TGF-β [56,57,58]. Blocking these receptors reverses TGF-β suppression on T cell proliferation, leading to enhanced immune response, reduced tumor growth, and decreased metastasis [57]. Galunisertib alone slowed bladder tumor growth, but when combined with αGD2 antibody, it nearly halted tumor progression by blocking TGF-β signaling and enhancing T cell production [45]. The reported side effects of Galunisertib that have emerged include nausea, fatigue, and hematological issues [58].

Bintrafusp Alfa is another TGF-β antagonist targeting bladder cancer cells. It has demonstrated efficacy not only in bladder cancer but also in cervical and triple-negative breast cancer. This bifunctional fusion protein combines TGF-βRII receptors with human immunoglobulin G1, blocking PD-L1 (programmed death-ligand 1) while “trapping” TGF-β [59]. This prevents TGF-β signaling in the tumor microenvironment (TME), leading to increased NK cell activation and reduced angiogenesis [60,61]. While generally well tolerated, side effects of Bintrafusp Alfa include itching, skin rashes, and decreased appetite [62].

### 4.2. IL-10

IL-10 is an anti-inflammatory cytokine secreted by TAMs, T cells, B cells, and NK cells. Within the TME, IL-10 activates signaling pathways that suppress the expression of pro-inflammatory cytokines [63,64]. Similarly to TGF-β, IL-10 can exert anti-tumor effects in limited amounts, but as tumors develop resistance, TAMs begin to overproduce IL-10. This overproduction alters N-glycan branching on surface glycoproteins, triggering the expression of Mgat5. The resulting glycosylation changes suppress the activation, cytokine production, and cytotoxic function of CD8+ T cells and NK cells, facilitating immune evasion, tumor progression, and metastasis in bladder cancer [65,66].

Pegilodecakin is a synthetic, pegylated recombinant IL-10, designed to mimic natural IL-10 but increase T cell proliferation [67]. It binds to IL-10 receptors and activates the JAK and STAT3 pathways, driving cytotoxic CD8+ T cell proliferation to boost the immune response and suppress tumor growth. [67,68,69]. Pegilodecakin is a newly developed drug with limited studies on its effects on bladder cancer; however, it shows promise in targeting IL-10 when combined with anti-PD-1 monoclonal antibody inhibitors across multiple types of cancer [70]. Side effects after the administration of this compound include red blood cell hemophagocytosis, fever, fatigue, injection site reactions, and maculopapular skin rashes [67].

### 4.3. IL-4

IL-4 is an anti-inflammatory cytokine primarily secreted by type 2 T helper cells (Th2), but can also be produced by TAMs, T cells, and NK cells in the TME [71]. IL-4 reduces inflammation by binding to IL-4 signal receptors in CD4+ T cells, activating the STAT6 pathways that differentiate them into Th2 cells which inhibit inflammatory cytotoxic CD8+ T cells [72]. This immune suppression allows bladder cancer to recruit Th2 cells and TAMs, increasing IL-4 secretion and further suppressing cytotoxic T cell production, promoting metastasis, immune evasion, and tumor growth [71,73].

Dupilumab, a monoclonal antibody originally designed to treat a wide range of inflammatory conditions, reduces the IL-4-induced pro-tumor phenotype of TAMs by binding to the IL-4 receptor alpha subunit (IL-4Rα) to block IL-4 signaling [74,75]. The impact of Dupilumab on cancer inhibition has not been established due to its recent development, but the IL-4 blockade mechanism shows promise for future immunotherapy in bladder cancer [74].

## 5. Engineered Cytokines, Fusion Proteins, and Combination Therapies

A deeper understanding of cytokine function in cancer immunotherapy reveals challenges such as a short half-life, low affinity, toxicity, poor tumor targeting, and unintended Treg activation [13]. Cytokine combination therapy helps overcome some of these limitations by employing cytokines in conjunction with other immunotherapeutic agents to enhance anti-tumor immune responses. By engaging multiple immune signaling pathways, this approach allows for broader immune activation and reduced risk of single-agent resistance [76,77,78,79]. Advancements in protein-engineering technologies have allowed for cytokine modifications that address additional barriers, improving their therapeutic potential [80]. The process involves designing the gene, purifying the cytokine, and testing its function to ensure it meets therapeutic goals [81]. A similar process is used to artificially complex two or more proteins together to form a fusion protein [82]. Cytokines can comprise one of the fusion protein subunits, making these engineered polymers relevant to immunotherapy. Chimeric cytokines broadly refer to cytokine–protein combinations, while more specific terms describe different engineered cytokines: supercytokines (enhanced cytokines), immunocytokines (cytokine–antibody fusion proteins), and engager cytokines (cytokine-bispecific immune engager fusion proteins) [80,83]. Factors to consider when engineering a cytokine for therapeutic use include the desired target, bioactivity and structure of the cytokine, structural format of the fusion protein, pharmacokinetics and pharmacodynamics, and dosing [84]. Here, we highlight treatments utilizing engineered cytokines, fusion proteins, and combination therapies that extend beyond strict pro- or anti-inflammatory functions.

### 5.1. EGF

EGF plays a critical role in cell proliferation, survival, and differentiation physiologically by activation of several intracellular signaling pathways such as RAS (Rat Sarcoma)/MAPK and PI3K (phosphoinositide 3-kinase)/AKT (protein kinase B) [85]. In bladder cancer, aberrant expression of the human EGF receptor 2 (HER2) has been strongly associated with tumorigenesis and poor prognosis. Targeting this dysregulation has become a growing focus of therapeutic research, with several phase II clinical trials conducted in 2023. One such trial combined the potent cytotoxicity of monomethyl auristatin E (MMAE) with the targeting capability of anti-HER2 antibodies to make the fusion protein disitamab vedotin (DV, RC48-ADC) for treatment of MIBC. DV achieved an ORR of 50.5% in HER2-positive UC, including subgroups with liver metastases and prior anti-PD-1/L1 therapy [86]. Another study combined trastuzumab-pkrb, a biosimilar of the monoclonal HER2 antibody trastuzumab, with the common chemotherapy drug paclitaxel, reporting a 48.1% ORR in patients with HER2-positive UC [87]. Additionally, the antibody–drug conjugate trastuzumab emtansine (T-DM1) showed a 38.5% ORR in a trial that was ultimately terminated early due to recruitment challenges [88]. Treatment-related adverse events (TRAEs) varied across studies, with the most common including peripheral neuropathy, neutropenia, and leukopenia [86,87]. Some patients discontinued treatment due to other unspecified TRAEs [88]. Despite these risks, all three studies reported significant clinical responses with manageable safety profiles [86,87,88].

### 5.2. CD40

CD40 is a receptor that binds to its ligand, CD40L, which is primarily expressed on activated T helper cells but can also be found on activated B cells and platelets. This interaction is essential for initiating both innate and adaptive immune responses as well as regulating inflammation, thrombosis, hematopoiesis, and tumor cell fate [89]. The human bladder TME shows enhanced CD40 expression, making it an ideal target to agonize for bladder cancer treatment. Anti-CD40 antibodies have a Fab and Fc domain and when the latter is engineered for enhanced affinity to its complementary receptor, the antibody’s agonistic activity is significantly increased. This Fc-enhanced anti-CD40 agonist antibody (2141-V11) was used for treatment in orthotopic murine bladder cancer models and achieved greater anti-tumor capabilities than both controls and BCG therapy through enhanced CD8+ T cell-dependent response. Systemic toxicity such as thrombocytopenia and transaminitis was avoided via intratumoral administration as opposed to systemic administration, contributing to 2141-V11’s progress onto clinical stages in treating solid metastatic tumors [90]. This was further supported by a study showing that the combination of 2141-V11 and IL-15 enhances efficacy in preclinical bladder cancer models, as evidenced by a reduction in tumor burden and an increase in activated CD8+ T cells within the TME [91]. A phase I trial that took place in 2024, however, combined the CD40 agonist MEDI5083 with durvalumab and showed only minimal efficacy (2.8% ORR) in advanced solid tumors with significant toxicity issues, including severe injection-site reactions and one death related to the side effects of MEDI5083 leading to the project’s termination despite its immunologic anti-tumor mechanism [92].

### 5.3. IL-15

IL-15 belongs to the same cytokine family as IL-2 and exhibits similar functions, including the activation of the innate and adaptive immunity pathways. It also acts as an anti-apoptotic factor for T cells and a stimulator of memory T cells, but unlike IL-2, it does not promote Treg proliferation. These roles would make it an ideal target for enhancement in cancer immunotherapy were it not for its instability and short half-life. To combat this undesirable trait, an IL-15 mutant (IL-15N72D) is complexed with a dimeric IL-15 receptor (15Rα Sushi domain) and an IgG1 Fc fusion to form N803. In murine models, N803 demonstrated a 35 times greater half-life than recombinant human IL-15 (rh-IL15), consequently prolonging the stimulation of CTLs and NK cells. In addition to there being no evidence of systemic toxicity in this treatment, significant anti-tumor activity was achieved when applied to murine tumor models, including in carcinogen-induced orthotopic NMIBC [93].

These research findings facilitated progress to the clinical application of N803 administration in conjunction with BCG. While BCG alone achieved a 50% response rate in NMIBC in a phase Ib clinical study, the combination therapy led to all patients remaining disease-free two years post-treatment, with no recurrence and intact bladders six years later. As a result, this study is now ongoing in phase II trials [94]. Another clinical study currently in phase II/III showed 100% survival at 24 months of follow-up for patients with BCG-unresponsive NMIBC [95]. These clinical findings led to a recent biologics license application being submitted to the FDA for the use of intravesical N803 in combination with BCG in NMIBC, which is now under review [4,93].

### 5.4. PD-1

PD-1 is a receptor expressed on activated T and B cells, while its ligands, PD-L1 and PD-L2, are found on epithelial cells during inflammation and on various antigen-presenting cells [96]. As an immune checkpoint, PD-1 signaling helps prevent excessive immune activation by inducing T cell exhaustion upon binding to its ligands, thereby reducing inflammation and preventing tissue damage [97]. Tumor cells exploit this mechanism by upregulating PD-L1 expression, leading to widespread T cell exhaustion. This immune suppression allows cancer cells to evade detection and proliferate unchecked [98].

Immune checkpoint blockade (ICB) therapy has emerged as a promising cancer treatment by inhibiting the PD-1/PD-L1 pathway [99]. Over the past decade, the FDA has approved several monoclonal antibodies targeting this pathway, including anti-PD-1 antibodies such as Nivolumab and Pembrolizumab, and anti-PD-L1 antibodies such as Atezolizumab, Durvalumab, and Avelumab. All three anti-PD-L1 antibodies have demonstrated immunosuppression and improved treatment outcomes specifically in urothelial cancer. Long-term efficacy is limited in these PD-1/PD-L1 therapies, leading to increased interest in combining ICB with other cytokine therapies [100,101].

Recent studies have highlighted the potential of combining the pro-immune mechanisms of IL-2 and anti-PD-1 therapy for improved anti-tumor outcomes. In one study, tumor-bearing mice were injected intraperitoneally with plasmids encoding an IL-2/anti-PD-1 fusion protein. Following the euthanasia of the mice, the analysis of tumor cells revealed decreased IL-2-related toxicity and increased tumor control. The researchers hypothesized that fusion proteins could be further engineered for specific target cells, including exhausted CD8+ T cells located within the tumor microenvironment [102,103]. A recent review compiling data on the combined treatment of IL-2 and anti-PD-1 showed positive potential for application in urothelial carcinoma, but more research is still needed [104].

### 5.5. IL-2 and IL-12

IL-2 and IL-12 both modulate immune responses and promote inflammation through distinct pathways. IL-2 stimulates T and NK cell proliferation, increasing the number of IFN-γ-secreting cells and their subsequent IFN-γ production [105]. In contrast, IL-12 is secreted by APCs to promote CD4+ T cell differentiation into Th1 cells and induces IFN-γ production in existing T and NK cells [106]. When combined, these two pathways demonstrate a greater efficacy in treatment than either pathway working independently. IL-2 upregulates IL-12Rβ1 and IL-12Rβ2, the subunits of the IL-12 receptor on NK cells, while IL-12 increases CD25 expression, the high-affinity IL-2 receptor. This creates a positive feedback loop that amplifies NK cell and CTL activation while simultaneously downregulating Treg expression [107,108].

Combined cytokine therapy was conducted with orthotopic bladder cancer in mice using a recombinant murine IL-12 and human IL-2. The treatment was administered via injection both intratumorally and subcutaneously at a distal site, with both cytokines given at suboptimal dosages to gauge the additive or synergistic effects of their tandem application. Both delivery sites demonstrated increased IFN-γ production, T cell cytotoxicity, and tumor cell elimination [109]. Another study reported severe gastrointestinal toxicity and epithelial cell apoptosis in mice being treated with the IL-2 and IL-12 combination therapy, revealing a significant challenge in the clinical adoption of this approach [110]. More recent research suggests that engineered cytokine variants may help mitigate these effects, offering a promising path forward [107].

### 5.6. Gene Therapy

Gene therapy is used in cancer treatment to enhance the body’s ability to recognize and eliminate tumor cells by introducing genetic material into targeted cells, typically tumor or immune cells [111]. This is achieved using viral vectors, most commonly adenoviral or lentiviral derivatives [112]. Transferred genes often encode immunostimulatory, costimulatory, or oncolytic factors, depending on the therapeutic goal. A phase I/IIa trial implemented gene therapy to treat bladder cancer by delivering the human CD40L gene via adenoviral vectors (AdCD40L) to stimulate systemic immunity. The first phase of this study was successful in gene transfer, heavy T cell infiltration in the bladder, reducing circulating Tregs, and reducing malignant cell load. The treatment was well tolerated with minimal side effects and showed promise early on for immunostimulating gene therapy in treating bladder cancer [113].

More recently, RBCEV have become an appealing alternative to viral vectors in gene therapy. RBCEVs derived from universal donor Type O red blood cells exhibit reduced immunogenicity and cytotoxicity compared to traditional viral vectors [114]. Using these vesicles as a vector for IL-12 delivery enhances tumor microenvironment targeting while minimizing dose-limiting toxicities. In a 2024 study, murine IL-12-encoding minicircle plasmids were isolated and loaded onto RBCEVs. Mice with MB49 bladder cancer received an intratumoral injection of the RBCEV vectors every two days, for a total of six doses. The satistical analysis revealed that the mice injected with the IL-12 minicircles exhibited a fivefold increase in IL-12 expression compared to the controls. Additionally, the kidney and liver function tests showed no observable increase in toxicity in the IL-12 minicircle group [43].

## 6. Discussion

Cytokine therapy shows promise in bladder cancer treatment by enhancing anti-tumor immunity through pro-inflammatory, anti-inflammatory, and engineered cytokines; fusion proteins; and combination therapies. While preclinical and early clinical studies demonstrate improved tumor targeting and elimination, challenges such as toxicity, immune suppression, and metastasis promotion remain.

The evidence included in this review has limited applicability to human treatment efficacy due to a lack of clinical studies compared to preclinical studies. The cited clinical studies primarily represent early-stage findings, and the complex interactions of cytokines within the tumor microenvironment are not yet fully understood. Additionally, the review process itself has inherent limitations, including potential publication bias favoring positive results and the risk of missing relevant data.

The implications for practice are currently limited as many therapies are still in development, but the success of treatments such as intravesical BCG and N803 suggest a future role for cytokine-based immunotherapies in bladder cancer treatment, especially for NMIBC. Future research should prioritize addressing risks such as toxicity and metastasis promotion while advancing preclinical findings through clinical trials.

## 7. Conclusions

An exploration into the mechanisms of cytokine therapies reveals innovative strategies to combat bladder cancer, including pro-inflammatory cytokines, anti-inflammatory cytokines, engineered cytokines, fusion proteins, and combination therapies, as summarized in Table 1. Strategies such as stimulating immune responses, improving targeting, and inhibiting immunosuppression demonstrate promise but also present challenges. While these therapies offer potential benefits such as tumor regression and enhanced immune activation, potential risks include toxicity and promoting metastasis. Despite these obstacles, the successes of cytokine therapies highlight the need for continued research to develop safe, effective, and minimally invasive treatments for bladder cancer.

## Figures and Tables

**Table 1:** Summary of cytokine therapies, detailing the target molecule, mechanism of action, treatment outcomes, and associated risks.

Cytokine Therapy	Molecule	Cytokine/Physiological Mechanism	Treatment Results	Treatment Risks	References
Pro-Inflammatory	IL-2	Promotes T cell proliferation and differentiation	Combined with BCG, enhances immune activation; IL-2 complexes reduce tumor size; BEMPEG conjunction therapy reduces tumor size	High doses: toxic; low doses: promotes immunosuppression	[18,19,20,21,22,23,24,25,26,27]
	IL-8	Activates neutrophils	Genistein suppressed IL-8; elevated IL-8 levels may be a biomarker	IL-8 promotes tumor angiogenesis and metastasis	[28,29,30,31,32,33,34,35,36]
	IL-12	Promotes IFN-γ production, increases NK cell activity, promotes Th1 differentiation, stimulates inflammation	CS/IL-12 resulted in 88% long-term survival; RBCEV delivery reduced tumor growth; M9241 and avelumab had little effect	Dose-dependent toxicity	[37,38,39,40,41,42,43,44]
Anti-Inflammatory	TGF-β	Regulates cell proliferation, suppresses immune cells, promotes angiogenesis	Galunisertib slowed tumor growth; Bintrafusp Alfa targeted cancer cells	Aids in cancer spread by suppressing immune cells; Galunisertib: nausea, fatigue, hematological issues; Bintrafusp Alfa: itching, skin rashes, decreased appetite	[51,52,53,54,55,56,57,58,59,60,61,62]
	IL-10	Blocks inflammatory cytokine expression, activates signaling pathways	Anti-tumor effects in small amounts; Pegilodecakin mimics natural IL-10 and increases T cell proliferation	Leads to tumor resistance and suppression of cytotoxic T and NK cell production, promoting immune evasion; Pegilodecakin: red blood cell hemophagocytosis, fever, fatigue	[63,64,65,66,67,68,69,70]
	IL-4	Reduces inflammation, suppresses cytotoxic T cells	Dupilumab reduces the pro-tumor phenotype of TAMs by blocking IL-4 signaling	Promotes metastasis, immune evasion, and tumor growth	[71,72,73,74]
Engineered Cytokine/Fusion Protein/Combination Therapy	EGF	Activates intracellular signaling pathways, promoting cell growth	DV achieved 50.5% ORR; trastuzumab-pkrb and paclitaxel achieved 48.1% ORR; T-DMI achieved 38.5% ORR	Peripheral neuropathy, neutropenia, and leukopenia	[85,86,87,88]
	CD40	Initiates innate and adaptive immune responses	2141-V11 supercytokine showed greater anti-tumor capabilities through enhanced CD8+ T cell response; 2141-V11 and IL-15 further enhanced tumor reduction and T cell activation	Systemic toxicity such as thrombocytopenia and transaminitis	[89,90,91,92]
	IL-15	Acts as an anti-apoptotic factor for T cells and a stimulator of memory T cells	N803 prolonged the stimulation of CTLs and NK cells and had significant anti-tumor activity; N803 and BCG achieved 100% survival at 24 months	No evidence of systemic toxicity	[4,93,94,95]
	PD-1	Prevents excessive immune activation by inducing T cell exhaustion	Combination with IL-2 demonstrated immunosuppression and improved treatment outcomes	Long-term efficacy is limited	[96,97,98,99,100,101,102,103,104]
	IL-2 and IL-12	Modulate immune responses and promote inflammation	Increased IFN-γ production, T cell cytotoxicity, and tumor cell elimination	Severe gastrointestinal toxicity and epithelial cell apoptosis	[105,106,107,108,109,110]
	Gene Therapy	Enhances the body’s ability to recognize and eliminate tumor cells	AdCD40L gene successfully transferred and increased T cells and decreased tumor load; IL-12-encoding plasmids loaded on RBCEVs showed fivefold increase in IL-12 expression	No observable toxicity	[43,111,112,113,114]

## Data Availability

The original contributions presented in this study are included in the article. Further inquiries can be directed to the corresponding author(s).

## Abbreviations

The following abbreviations are used in this manuscript:

AKT	Protein kinase B
APC	Antigen-presenting cell
BCG	Bacillus Calmette–Guérin
CAR T	Chimeric antigen receptor T cell
CTLs	Cytotoxic T cells
CD40	Cluster of differentiation 40
CD40L	CD40 ligand
DV	Disitamab vedotin
EGF	Epidermal growth factor
EMT	Epithelial–mesenchymal transition
ERK	Extracellular signal-regulated kinase
FAK	Focal adhesion kinase
GM-CSF	Granulocyte–macrophage colony-stimulating factor
HER2	Human epidermal growth factor receptor 2
ICB	Immune checkpoint blockade
IFN	Interferon
IL	Interleukin
IL-2	Interleukin-2
IL-2c	IL-2 complex
JAK	Janus kinase
MIBC	Muscle-invasive bladder cancer
MAPK	Mitogen-activated protein kinase
MMAE	Monomethyl auristatin E
NMIBC	Non-muscle-invasive bladder cancer
NK cells	Natural killer cells
ORR	Objective response rate
PAMP	Pathogen-associated molecular pattern
PD-1	Programmed death receptor-1
PD-L1	Programmed death-ligand 1
PI3K	Phosphoinositide 3-kinase
RAS	Rat Sarcoma
RBCEVs	Red blood cell-derived extracellular vesicles
rh-IL-15	Recombinant human IL-15
STAT	Signal transducer and activator of transcription
TAMs	Tumor-associated macrophages
T-DM1	Trastuzumab emtansine
TGF-β	Transforming growth factor-β
Th1	Type 1 T helper cells
Th2	Type 2 T helper cells
TME	Tumor microenvironment
TNFs	Tumor necrosis factors
Tregs	Regulatory T cells
UC	Urothelial cancer
